# Vertebral Fractures in Pediatric Suicidal Jumpers: A Retrospective Study with Epidemiological and Clinical Analysis before and after the COVID-19 Pandemic

**DOI:** 10.3390/jcm12237412

**Published:** 2023-11-29

**Authors:** Marco Crostelli, Osvaldo Mazza, Francesca Manfroni, Federico Tundo, Valeria Calogero, Marianna Mazza, Roberto Averna, Stefano Vicari

**Affiliations:** 1 Spine Surgery Department, Bambino Gesù Children’s Hospital IRCCS, 00168 Rome, Italy; marco.crostelli@opbg.net (M.C.); francesca.manfroni@opbg.net (F.M.); federico.tundo@opbg.net (F.T.); valeria.calogero@opbg.net (V.C.); 2 Institute of Psychiatry and Psychology, Fondazione Policlinico Universitario A. Gemelli IRCCS, Università Cattolica del Sacro Cuore, 00168 Rome, Italy; marianna.mazza@policlinicogemelli.it (M.M.); stefano.vicari@opbg.net (S.V.); 3 Childhood and Adolescent Neuropsychiatric Unit, Neuroscience Department, Bambino Gesù Children’s Hospital IRCCS, 00168 Rome, Italy; roberto.averna@opbg.net

**Keywords:** pediatric spine fractures, suicidal jumpers, spine fracture surgery, COVID-19, suicidal attempts

## Abstract

Background: From the beginning of the COVID-19 pandemic, reports in the literature confirm a significant increase in suicide attempts in children and adolescents. At the Bambino Gesù Pediatric Hospital Emergency Department (Rome, Italy), there was a dramatic increase in suicidal jumpers. Many of these presented vertebral fractures. Methods: This retrospective study includes all suicidal jumpers with vertebral fractures treated from April 2017 to March 2023. We collected and compared data from three years before to three years after the pandemic, analyzing vertebral fractures. Results: From April 2019 to March 2020, 141 cases of suicide attempt arrived at the emergency department. Five of these were suicidal jumpers without vertebral fractures. From April 2020 to March 2023, 362 cases of suicide were hospitalized and 19 were suicidal jumpers; 12 reported vertebral fractures (mean age 14 years). Seven patients were treated by percutaneous pedicle fixation. Three patients needed an open spinal surgery by posterior approach. One case with cervical fractures was treated by Halo-Vest. Conclusions: This is the first report that shows a sharp increase in vertebral body fractures due to suicide jumping attempts in children and adolescents. This could be a new epidemiological phenomenon persisting or even increasing over time in the pediatric population as a consequence of the COVID-19 pandemic.

## 1. Introduction

Suicide is a relatively rare event in pediatric population, but its prevalence increases during adolescence and it is highly lethal [1]. The World Health Organization states that suicide is a social problem, and prevention is particularly important for managing suicide attempts in the youngest population, which has longest life expectancy [2]. According to the World Health Organization, suicide is the fourth leading cause of death among 15- to 29-year-olds [2] and the United Nations International Children’s Emergency Fund in “The State of the World’s Children 2021” highlights that suicide is the second leading cause of death among 15- to 19-year-olds in Europe [3]. Almost 1200 youth between the ages of 10 and 19 years die by suicide every year in Europe [3]. According to the Centers for Disease Control and Prevention (CDC), 2018 suicide is the second most common cause of death among 10- to 14-year-olds and 20- to 34-year-olds in USA [4]. Suicide in youths is globally recognized as an important public health problem [2].

Poisoning by pesticides represents the 20% of suicides in low- and middle-income countries [2], but there are large differences between countries and genders in suicide attempt methods [5,6]. In an analysis of 15 European countries, Värnik et al. [6] claimed that hanging and jumping from height were, respectively, the first and the second suicide method for both genders in people between 15 and 24 years of age, followed by using a moving vehicle for males and poisoning for females. According to Kolves et al., the most frequent suicide method in children and adolescent aged 10–19 years in different countries worldwide is hanging. Jumping from height is also an important alternative method [5].

Many recent studies reported in the literature confirm a significant increase in mental health disease in children and adolescents from the beginning of the COVID-19 pandemic, with a rise in suicidal ideation and suicide attempts [7,8,9,10,11,12,13,14,15,16,17].

From the beginning of the COVID-19 pandemic, admission of patients with mood disorders, self-injurious behaviors, and suicidal ideation significantly increased in our hospital [16]. We noted a significant increase in suicidal attempts by jumping from height in adolescent patients, compared with pre-COVID data. Many of these suicidal jumper attempts resulted in vertebral fractures.

There are few studies on the type of injuries associated with falls from heights in pediatric patients. In general, traumatic spinal injuries in the pediatric population are uncommon and are frequently related to motor vehicle crashes [18,19]. In patients younger than 4 years in age, most vertebral fractures involve the upper cervical spine (C0–C4) [20], and this is due to anatomical age-related conditions. Thoracic spine is the most common localization of spine fractures in the general pediatric population, followed by lumbar spine [21,22]. Conservative treatment, for 6 to 8 weeks with a thoraco-lumbo-sacral orthosis, is the gold standard for stable fractures [21,23]. Neurological impairment is reported in about 5% of cases [21] and is considered a risk factor for progressive spinal deformity [23].

The care of vertebral fractures in suicidal jumpers is peculiar and must consider the issue of patients’ mental health.

This work highlights the new and widespread epidemiological phenomenon of a high frequency of suicidal attempts with an uncommon modality in adolescents following the COVID-19 pandemic. It is particularly important to analyze the treatment of numerous complex vertebral fractures in polytrauma pediatric patients who committed suicide attempts by jumping from heights.

## 2. Material and Methods

In this retrospective study, we reviewed all patients treated at Bambino Gesù Children’s Hospital Emergency Department in Rome from 1 April 2017 to 31 March 2023 for suicidal attempt.

We compared the three-year post-COVID 19 pandemic beginning with the three-year period before pandemic. Italian government imposed a national lockdown on 9 March 2020, but in this study we decided to start the pandemic period from 1 April, about 1 month after, to better highlights the effects.

In our hypothesis, the dramatic increase in suicidal attempts by jumping from height within the pediatric and adolescent population in Italy, and consequent increase in incidence of vertebral fractures, is the direct consequence of pandemic restrictions and limitations of everyday life and normal social interaction on the mental health of the youngest part of the Italian population.

To our knowledge, this is the first work specifically focusing on the increase in the occurrence of suicidal jumpers’ vertebral fractures within the pediatric population during the COVID-19 pandemic.

Only patients with vertebral fractures for a suicidal attempt were included. Fracture classification, treatment, and midterm follow-up were analyzed.

Thoracolumbar, subaxial cervical, and sacropelvic fractures were classified according to AO spine [24,25,26] and cervical fractures were classified using the Anderson–D’Alonso classification [27].

The fracture pattern in these jumpers is not different from other nonsuicidal high-energy spine fractures treated in patients of similar age, but particular consideration has been given to the choice of treatment, which to some extent is different from the treatment for patients without suicidal attempt issues. We decided treatment according to the grade of fracture instability, neurological impairment, and psychiatric treatment needed. Usually, patients who attempted suicide require immediate and prolonged psychiatric treatment with a long hospitalization period in dedicated wards. Since the possibility of early mobilization of the patient without orthosis is paramount to begin successful psychiatric rehabilitation treatment, in many “borderline” spine fractures (vertebral fractures without neurological impairment that in a patient without psychiatric issues could be treated with a prolonged orthosis or cast immobilization), we opted for percutaneous surgical treatment to avoid long bracing treatment.

The study received approval from the local ethical committee (Ethics Committee of the Bambino Gesù Pediatric Hospital, Rome, Italy, ID 2426-OPBG-2021). Written informed consent was obtained from all participants and/or their parents for minors.

## 3. Results

At Bambino Gesù Children Hospital, there were 141 cases of suicidal attempts from 1 April 2017 to 31 March 2020 and 5 (2.8%) of these were suicidal jumpers; none of them reported vertebral fractures.

From 1 April 2020 to 31 March 2023, there were 362 suicidal attempts with 19 (5.2%) suicidal jumpers. Twelve (63.2%) of the suicidal jumpers had a vertebral fracture (M:F = 4:8) (Table 1).

The mean age was 14 years (range 10 to 16). Only two patients had a neuropsychiatric diagnosis before the suicide attempt (16.6%). The most common level of fracture was L1 (six cases). Thoracic spine was involved in four cases and lumbar spine in ten. Hospitalization period ranged from 12 to 210 days, with high variability mostly due to neuropsychiatric care.

Vertebral fractures are a direct consequence of the impact of jumpers’ bodies with soil. Owing to the pandemic lockdown restrictions in Italy, data for suicidal attempt circumstances from first emergency teams that rescued patients are not available; thus, it is not possible to determine a relationship between the height of the jump or nature of the impact surface and severity or pattern of fractures.

According to AO spine thoracolumbar classification [24], there were five A0, ten A1, three A3, and five A4 vertebral fractures, alone or in association (Table 1). Moreover, there was an “U shaped sacral fracture” (C3 AO spine) [26], a transforaminal sacral fracture (B3 AO spine) [26], a coccyx fracture (A1 AO spine) [26] and an Anderson–D’Alonso C2 type II fracture [27].

In the latter case, a burst C5 fracture (A4 AO spine) and a compression C6 fracture (A1 AO spine) [25] were associated with the C2 fracture and she was conservatively treated with a Halo-Vest for 3 months.

**Figure 1:**
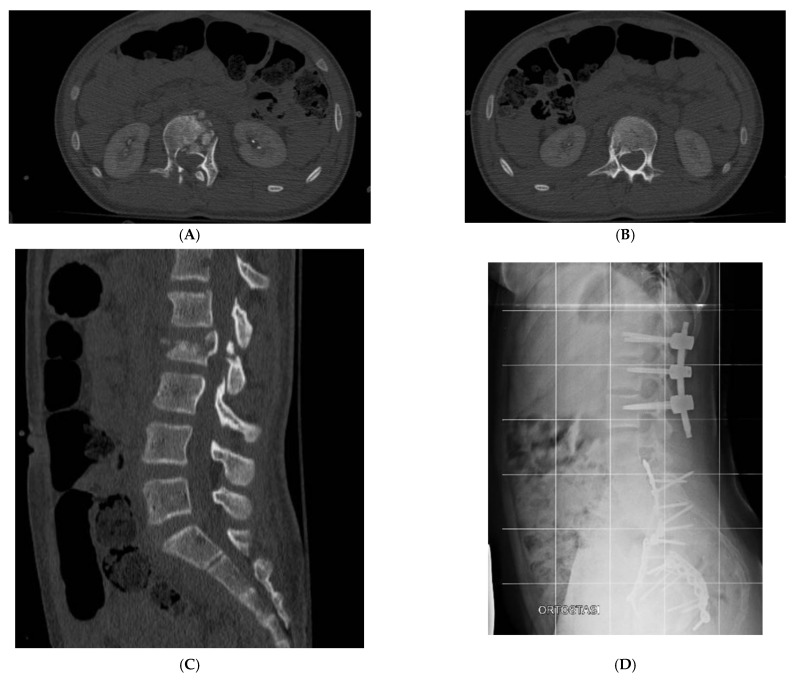

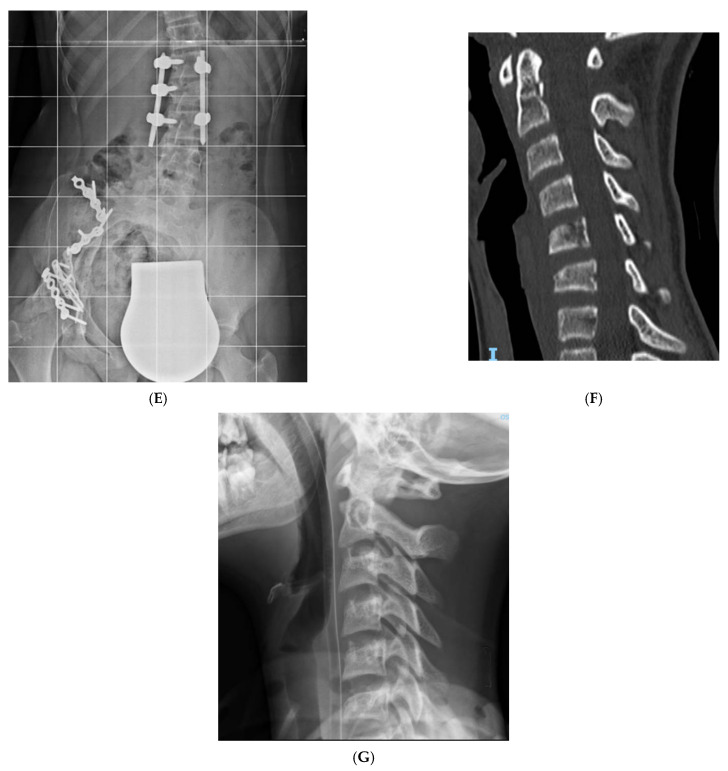
Case 6 (Table 1): (**A**,**B**) preoperative axial CT scan of L1 and L2; (**C**) preoperative sagittal CT scan of lumbar spine; (**D**,**E**) anteroposterior and lateral X-ray of lumbar spine after surgery; (**F**) sagittal CT scan of post-traumatic cervical spine; (**G**) X-ray lateral view in Halo-Vest.

**Figure 2:**
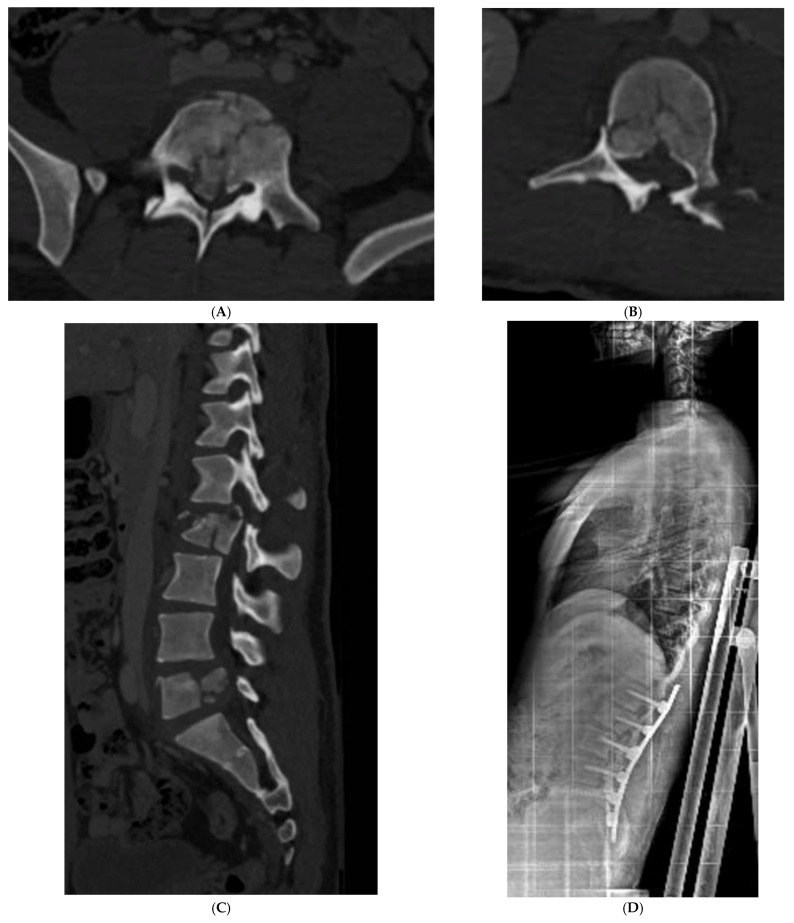
Case 10 (Table 1): (**A**) preoperative axial CT scan of L5; (**B**) preoperative axial CT scan of L2; (**C**) preoperative sagittal CT scan of lumbar spine; (**D**) postoperative X-ray lateral view in sitting position.

Only two patients with thoracolumbar A0 and A1 types of fractures (cases 4 and 5, Table 1) were conservatively treated with a rigid thoracolumbar orthosis for 3 months with good compliance and results. Percutaneous pedicle fixation was applied in seven patients with an indirect decompression and to restore and improve sagittal balance (Figure 1 and Figure 2; Table 1, case 6 and 10). Only in two cases we observed neurological impairment. In both cases, an open spinal decompression and posterior spinal fusion was performed. All surgically treated patients used a soft spine orthosis for 1 month to facilitate early mobilization and to reduce muscular pain.

All patients had other appendicular fractures or other injuries that in some cases needed surgical treatment. Eight patients underwent other procedures: seven orthopedic surgeries for appendicular fractures, one maxillofacial surgery for an orbital floor fracture, and one vascular and one proctological surgery. Many different medical specialties were involved in these patients’ care.

Four patients had preoperative neurologic deficits. The first patient (case 1, Table 1) presented with a diffuse axonal injury and a left hemiparesis that completely recovered in 4 months. The second (case 3, Table 1) had a transitory right peroneal nerve palsy. The third (case 6, Table 1) had a paraplegia and complete neurogenic bowel and bladder dysfunction; the latter had a complete neurogenic bowel and bladder dysfunction which improved during the last month (case 12, Table 1).

At last follow-up, the third patient showed a persistent complete sphincter deficiency and a partial recovery of paraplegia. Nowadays, she can walk with crutches for short distances. Otherwise, the latter patient only has a urinary retention.

One year after trauma, four patients underwent implant removal surgery (cases 1, 2, 3, and 8, Table 1) with excellent clinical and radiological results. All these patients resumed their daily activities, including sports.

## 4. Discussion

Recent literature highlights concern about the consequences of the COVID-19 pandemic on pediatric population mental health [7,8,9,10,11,12,13,14,15,16]. In this study, the comparison between 3 years before and 3 years after pandemic demonstrates an increase in suicidal attempts. Comparing the two periods, suicidal attempts after the pandemic increased by 61.1% and suicidal jumpers increased by 73.7%. In the 3 years before the pandemic, no vertebral fractures in suicidal jumpers were noted. Otherwise, in the other 3 years analyzed, during the pandemic, 63.2% of suicidal jumpers reported vertebral fractures.

The purpose of our study was to analyze this new and dramatic phenomenon and, in particular, to report the peculiarity of vertebral fracture treatment in young suicidal jumpers during the pandemic.

To our knowledge, this is the first study that shows a significant increase in suicide jumping attempts and a related increase in vertebral fractures in pediatric patients after the pandemic.

Regarding the twelve patients included in the study and according to concerns reported in the literature on the increase in neuropsychiatric diseases, only 16.6% had a previous neuropsychiatric diagnosis. Even if it is not statistically significant, considering the limited timeframe and low number of patients involved, in our opinion, these raw data show the devastating effect of the COVID-19 crisis on the social environment and behavioral habits of youngest Italian population.

Before the COVID-19 pandemic, jumping from heights was an unusual suicide attempt behavior in our country’s adolescent population, and experience with surgical treatment of spine fractures in adolescent patients involved in these suicidal attempts was almost inexistent.

In this study, different types of vertebral fractures were found and most were surgically treated (83.3%). The pattern of fractures presented in this study are similar to high-energy spine fractures due to other kinds of injuries (e.g., motor vehicle crash), but their frequent recurrence in a relatively short period of time is a new finding. Some of the fractures treated are rare in the pediatric population (i.e., spinopelvic dissociation) and are more frequent in adults who jumped from heights. Sacral fractures are about 0.16% of all pediatric trauma [28] and cervical fractures are also rare and different according to age [29]. Only 1.5% of pediatric trauma cause cervical spine injury, but 60 to 80% of vertebral pediatric injuries involve the cervical spine [29].

In the literature, conservative treatment with orthosis for 6 to 8 weeks is suggested for stable vertebral fractures without neurologic impairment [21,23]. Nonsurgical treatment is also indicated in the case of burst fractures without neurologic impairment, since no significant differences have been demonstrated in functional outcomes compared to operative treatment [30,31].

In this study, surgery was performed not only to decompress or to restore a better sagittal balance but also to achieve a faster recovery. Conservative spine treatment with rigid orthosis or casts for a long period following a suicide attempt could be an additional burden for these patients. Surgical percutaneous treatment for thoracolumbar fractures allows early mobilization, and this can ensure to the patient a quick resumption of daily activities. Suicidal jumper patients are usually adolescents, with multiple injuries and severe mental health diseases, who face long periods of psychiatric and multidisciplinary treatments. Surgery can be considered a temporary internal fixation as an alternative to conservative treatment with a rigid orthosis, which could be a hitch to the patient’s therapeutic path.

However, cervical spine surgery is much more invasive and the only case with multiple cervical fractures and good results was conservatively treated with a Halo-Vest. In this patient, neurological impairment was not observed, and a surgical treatment had no real advantage over conservative treatment to obtain better fracture reduction and stabilization. In this case, surgery could have a potential higher risk of complications (i.e., surgical site infection, vascular and nervous iatrogenic damage, blood loss) and instrumentation spread on more levels. Patients and relatives occasionally experience difficulty in complying with Halo-Vest treatment, even if Halo is somehow preferable to collar bracing or casting; during long treatment, patient discomfort is less when using Halo-Vest, particularly regarding alimentation and hygiene. In our case, patient and parents showed good compliance with Halo-Vest, and the orthopedic treatment did not impair her psychiatric therapy.

In this study, four patients had neurologic lesions and two with severe outcomes. Treatment of these patients was challenging, especially when they were isolated from relatives and friends. Moreover, spinal treatments had to be coordinated with the need for treatment of other associated injuries. A multidisciplinary team has been essential to optimize the management of these patients and avoid major complications.

The main limitation of this study is the low number of involved patients; a multicenter study at European level, where there were similar pandemic conditions and restrictions, could certainly widen the cohort of patients with the aim to define the best treatment guidelines.

In future research, we could expand these preliminary results in order to clarify if this dramatic phenomenon and related injuries will remain as a grim result of postpandemic changes in social behavior or if suicidal jumper attempts in adolescents will return to prepandemic incidence.

## 5. Conclusions

This study shows an exponential increase in pediatric vertebral fractures due to suicide jumping attempts after the COVID-19 pandemic. These are high-energy fractures that are rather unusual in adolescents, and rarely observed in specific cases (such as spinopelvic dissociation). The unusual high frequency of suicidal attempts with an uncommon modality in adolescents is a new epidemiological phenomenon after the COVID-19 pandemic. The high number of complex vertebral fractures, in polytrauma pediatric patients who committed a self-harm act, could be a big issue for a spine surgeon. The choice of treatment must be guided by biological and biomechanical knowledge, but also by the mental health condition of young patients and by other necessary treatments. In our experience, percutaneous surgery was the treatment strategy used most and allowed for fast recovery. Suicidal jumper patients must be handled by a multidisciplinary team to optimize and personalize therapeutic management. In future studies, we could clarify if suicidal jumper attempts in adolescents will return to prepandemic incidence or, conversely, if this dramatic phenomenon will remain as a long-term consequence of COVID-19 pandemic.

## Figures and Tables

**Table 1:** Patients involved in the study.

	Patient	Age	Sex	Vertebral Fracture—AO Classification	Other Fractures	Other Injuries	Spine Treatment	Hospitalization Days	Follow-Up
1	V.C.G.C.	15 y 9 m	F	L1 (A4), L4 (A1), Coccyx (A0)	right distal tibia, right fibula, left proximal humerus, left ulnar styloid process	pneumothorax, splenic injury, subdural hematoma, diffuse axonal injury	T12 to L2 Percutaneous pedicle fixation	118	22 months No deficit Implant removal
2	**A.M.**	14 y 1 m	M	L1 (A3)	right ankle		T12 to L2 Percutaneous pedicle fixation	20	21 months No deficit Implant removal
3	Z.P.E.	14 y 11 m	M	L3 (A1), L4(A4)	right acetabulum, right ischiopubic branch, right tibia, bilateral wrist, bilateral heels	pneumothorax, aortic isthmus lesion, right peroneal nerve injury	L3 to L5 Percutaneous pedicle fixation	37	17 months No deficit Implant removal
4	B.T.M.A	12 y 7 m	F	D11 (A1)	multiple ribs		Conservative	15	17 months No deficit
5	R.S.M.	16 y 1 m	F	D8 (A0), D11 (A1), L1 (A1), L4 (A1)	right ankle, left distal radius		Conservative	12	11 months No deficit
6	L.G.G.	10 y 5 m	F	C2 (AD 2), C5 (A4), C6 (A1), L1 (A1), L2 (A3), L3 (A0), L4 (A0), Right sacroiliac joint	multiple ribs, right acetabulum, ilio-ischiopubic bilateral branch, left heel	liver injury, splenic injury, pleural effusion	Halo-Vest and L1 to L3 Percutaneous pedicle fixation	64	10 months No deficit
7	H.B.	15 y 5 m	F	L5 (A0), Sacral U-Shaped Fracture (C3)	right acetabulum, bilateral olecranos, right femural shaft	paraplegia, complete neurogenic bowel and bladder dysfunction	L4 to Pelvis posterior open fusion, left sacral plate and L4 to S1 decompression	30	8 months complete neurogenic bowel and bladder dysfunction; motor deficit of the right limb partial recovered
8	**D.G.**	12 y 10 m	F	D6 (A1), D7 (A1)	multiple right ribs	pneumothorax	D5 to D8 Percutaneous pedicle fixation	210	9 months No deficit Implant removal
9	M.D.	16 y 5 m	M	D12 (A1), L1 (A3)		anal injury	T10 to L2 Percutaneous pedicle fixation	56	6 months No deficit
10	G.A.	16 y 6 m	M	L2 (A4), L5 (A4)	right orbital maxilla surface, right open tibia and fibula		L1 to S1 Percutaneous pedicle fixation	40	6 months No deficit
11	B.V.	15 y 3 m	F	L1 (A4)		pulmonary contusion, complete neurogenic bowel and bladder dysfunction	T11 to L3 open pedicle screw fixation and posterior L1 decompression	14	1 month Urinary retension
12	P.E.	12 y	F	L5 (A0), Sacral fracture (B3)	orbital floor, bilateral nose bones, ilio-ischiopubic left branch, pubic symphysis diastasis		L4 to ilium left open fixation and iliosacral screw	33	1 month No deficit

Legend: Bold text for patients with previous neuropsychiatric diagnosis. Underlined text is for lesions surgically treated. A, B, and C (AO classification), AD (Anderson–d‘Alonso classification).

## Data Availability

Data are unavailable due to privacy restrictions.
